# Occurrence of Post-Transplant Lymphoproliferative Disease in a Renal Transplant Patient

**DOI:** 10.7759/cureus.17930

**Published:** 2021-09-13

**Authors:** Sofiah Ali, Nahel Haji, Priyanjali Pulipati, Emad A Wahashi, Kalyana Ramamurthi

**Affiliations:** 1 Internal Medicine, St. Joseph Mercy Oakland, Pontiac, USA; 2 Internal Medicine, St. Joseph Mercy Oakland Hospital, Pontiac , USA; 3 Internal Medicine, St. Joseph Mercy Oakland Hospital, Pontiac, USA; 4 Nephrology, St. Joseph Mercy Oakland Hospital, Pontiac, USA

**Keywords:** transplant nephrology, t-cell immunosuppression, post-transplant lymphoproliferative disorders, hodgkin's lymphoma non-hodgkin's lymphoma, epstein-barr virus, malignant ascites, deceased donor kidney transplant

## Abstract

Post-transplant lymphoproliferative diseases (PTLD) are a group of lymphoid disorders that occur in the setting of solid organ or hematopoietic transplantation. Risk factors for the development of PTLD include type of organ transplanted, degree/duration of T-cell immunosuppression, and Epstein-Barr virus (EBV) status. After initial infection, EBV lies dormant in memory B-cells and persists at low levels throughout the lifetime. In an environment of chronic T-cell immunosuppression, the underlying EBV infection remains uncontrolled, resulting in malignant B-cell lymphoproliferations that causes PTLD.

While PTLD is the most common malignancy associated with solid organ transplants, they are a serious complication and require a low threshold of suspicion for diagnosis. Oftentimes symptoms are nonspecific, such as weight loss and malaise, and many patients present without associated lymphadenopathy.

We present the case of a 30-year-old Asian American female who developed PTLD, specifically large B-cell non-Hodgkin's lymphoma, five years after receiving a deceased-donor renal transplant.

## Introduction

While PTLD can be Epstein-Barr virus (EBV) negative or positive, EBV-positive lymphoma is much more acute, rare, and life-threatening [1]. The condition is a direct consequence of EBV-infected B-cell proliferation in patients with suppressed and/or depleted T-cells. Data suggests that, at the time of transplantation, EBV-seronegative recipients are 24 times more likely to develop PTLD than their EBV-seropositive counterparts [2].Once the body acquires an EBV infection, it is the role of T-cells to keep it under control during intermittent EBV-lytic phases; therefore, T-cell suppression plays a significant role in the development of EBV-positive PTLD [3]. In these patients, the unopposed EBV-infected B-cells begin to hyper-proliferate, leading to lymphoma. 

Risk factors for the development of PTLD vary depending on the intensity and duration of immunosuppressive therapy, as well as the type of organ that is transplanted [4]. EBV-infected B-cells that give rise to PTLD can originate either from the host (transplant recipient) or the donor following solid organ transplantation. Host-derived PTLD is more common and oftentimes a multisystem disease, in contrast to donor-derived PTLD which tends to be limited to the allograft tissue [1]. 

PTLD in solid organ transplants occurs with the highest incidence in intestinal and multi organ transplants (5 - 20%), followed by lung and heart transplants at 2 - 10%, and finally renal and liver transplants at 1 - 5% [5].The cumulative incidence of PTLD in renal transplant patients is 1 - 3% with more than 80% of cases occurring in the first year after transplantation [6-8]. Our case report presents a unique patient; one who not only develops PTLD several years after transplantation, but in an organ that typically has the lowest possible incidence of the disease.

## Case presentation

We present the case of a 30-year-old Asian American female with a history of end-stage renal disease (ESRD) due to IgA nephropathy, status-post deceased-donor renal transplant (DDRT) in 2013. This graft failed due to chronic allograft dysfunction in 2015 secondary to BK virus associated nephropathy (BKVAN) complicated by antibody mediated rejection. In 2016, she received a second DDRT whose cytomegalovirus (CMV) and Epstein-Barr virus (EBV) status was as follows: CMV donor negative/recipient positive and EBV recipient positive.

She presented to the emergency department in May 2021 with severe abdominal distention, pain, and intractable vomiting. She was noted to have a 17-pound weight loss over the last six months. At the time of admission, vital signs were stable other than an elevated heart rate of 120 beats per minute. Initial labs revealed creatinine of 1.43 (baseline), calcium of 11.1, white blood cell count of 13.8, and erythrocyte sedimentation rate (ESR) of 49 (reference range: 0 - 20 mm/hr). Physical exam was significant for abdominal ascites, with a positive fluid wave.

Computed tomography (CT) abdomen with and without contrast showed peritoneal nodularity, stranding, lymphadenopathy, and omental thickening diffusely throughout the abdomen with small perihepatic ascites. No hydronephrosis was noted. At the time, the patient was compliant on her medication regimen of tacrolimus 8mg in the morning and 9mg at bedtime, along with azathioprine (150mg daily), and prednisone (10mg daily). A recent tacrolimus trough level taken before admission was 6.9 (reference range: 5 - 15 ng/mL).

**Figure 1:**
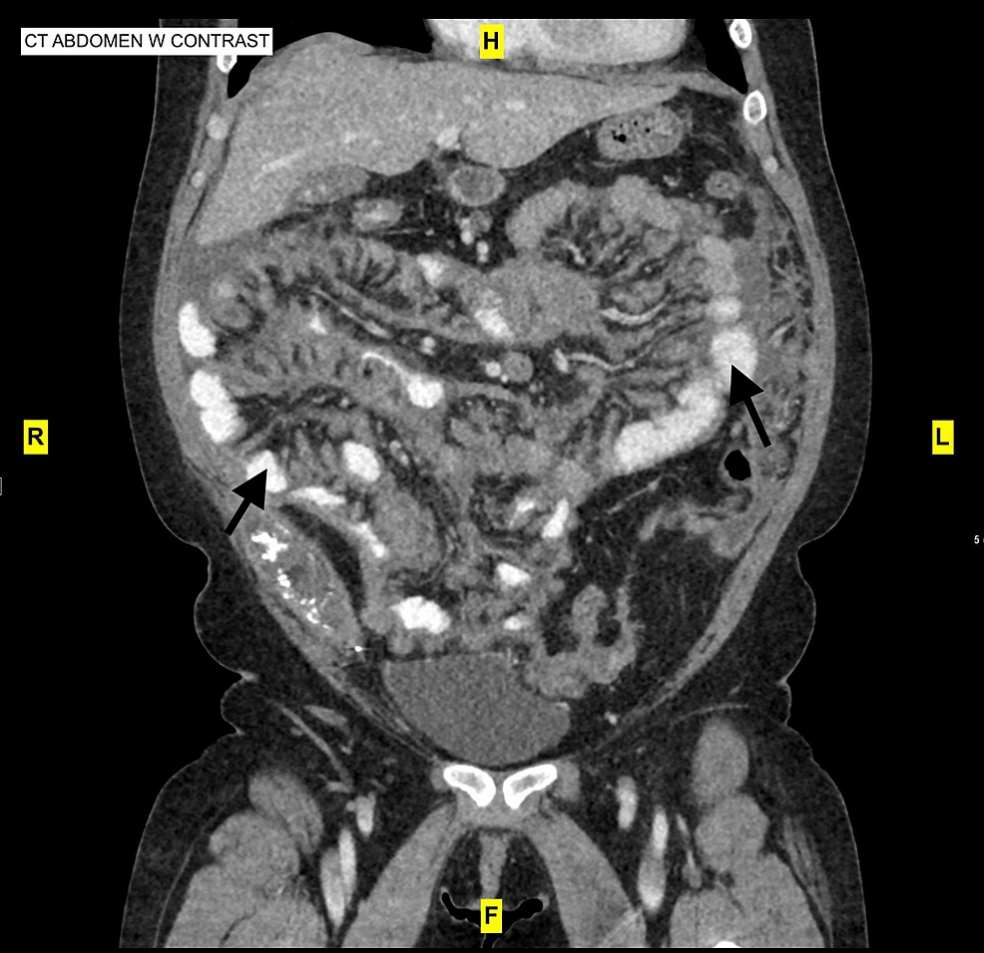
CT abdomen with contrast showing peritoneal nodularity, stranding, and omental thickening diffusely throughout the abdomen with small perihepatic ascites

Therefore, the CT findings seemed concerning for lymphoproliferative disease in the setting of tacrolimus. Sagittal view of CT abdomen with contrast is shown in Figure 1. 

CT pelvis was obtained to rule out primary pelvic pathology, however results were inconclusive. A pelvic ultrasound followed which depicted nonspecific abnormal ovaries with diffuse enlargement and hypoechogenicity, possibly due to vascular compromise, however overt ovarian malignant pathology was not identified. Abdominal paracentesis was suggested for further information regarding lymphoproliferative malignancy. 

On Day 2 of hospital admission, paracentesis was performed; 1L of bloody fluid was removed and analyzed, showing greater than 300,000 red blood cells and 200,000 white blood cells; ascitic fluid/serum ratio was 18. Further body fluid analysis showed elevated lactate dehydrogenase (LDH) of 4849 and cancer antigen (CA-125) of 161, suggesting either an inflammatory or malignant process. Cancer antigen (CA) 19-9 was normal at 13.4 and carcinoembryonic antigen (CEA) was normal at 0.6. Erythrocyte sedimentation rate (ESR) continued to rise to 56 mm/hr, although a C-reactive protein (CRP) level was not drawn. Initial fluid cytology showed atypical single cells associated with marked cellular degenerating changes and inflammation. Ascitic fluid showed CD45 negative, and CD20 positive cells (but the latter was more reactive in nature.) 

On Day 4, the patient was transferred to a nearby hospital, where her primary transplant team was located, to maintain continuity of care. A repeat paracentesis and fluid cytology was positive for malignant cells, CD20 positive cells, and was also noted to have blood cells consistent with blood cell lymphoma. The differential at the time included diffuse large B-cell vs. Burkitt’s lymphoma consistent with PTLD; tissue pathology from the omental lesions, however, was required for a confirmed final diagnosis. 

Six days after the patient was admitted to the secondary hospital, she left against medical advice. She presented on the same day, once again, to our Emergency Department, complaining of worsening abdominal pain and distension. The patient underwent therapeutic paracentesis where 3.7L of cloudy, serosanguinous fluid was removed. Fluid studies were consistent with spontaneous bacterial peritonitis (SBP), and so ceftriaxone was started, prednisone was held, and tacrolimus was continued.

Over the next few days, the patient’s condition significantly worsened; she began having hematemesis, worsening lactic acidosis, and tachycardia. She was transferred to the intensive care unit where hemodialysis (HD) was started due to worsening renal function.

The patient became progressively hypotensive overnight, requiring vasopressors. Her abdominal distension worsened further, and she began to experience respiratory distress secondary to abdominal pain. Uric acid levels were elevated at 11.8 and so rasburicase for tumor lysis syndrome (TLS) vs. acute urate nephropathy was initiated. Azathioprine was stopped due to initiation of allopurinol 100mg daily for hyperuricemia. At this point tacrolimus was held. The following day (Day 5 of her latest admission) the patient was intubated and started on continuous renal replacement therapy (CRRT) secondary to persistent hyperkalemia refractory to medical treatment. 

During the entire course of hospitalization, the patient’s blood cultures, and peritoneal fungal cultures were consistently negative. 

A final pathology report on the most recent ascitic fluid analysis and cytology returned and showed “findings consistent with a B-cell non-Hodgkin’s lymphoma (NHL) with a large cell component.” Further classification would require a tissue biopsy of an affected area along with flow cytometry and cytogenetic studies.

At this point, the patient’s EBV titers returned: results are shown in Table 1. 

**Table 1:** BK, CMV, and EBV viral level test results BK Virus: Polyoma BK virus; CMV: Cytomegalovirus; EBV EA: Epstein-Barr virus, early antigen; EBV VCA: Epstein-Barr virus, viral capsid antigen; PCR: polymerase chain reaction

Viral Tests		
BK Virus DNA Qualitative Plasma	Not Detected	Reference Range: Not Detected
BK Quantitation	< 125	Reference Range: < 125 cy/mL (clean yield/mL)
Log BK Virus DNA, Plasma	< 2.10	Reference Range: < 2.10 Log cy
CMV DNA Qualitative PCR	Not Detected	Reference Range: Not Detected
CMV Quantitative DNA PCR	< 50	Reference Range: < 50 IU/mL (International Units/mL)
CMV DNA (Centipoise (cP)/mL)	< 126	Reference Range: < 126 cy/mL
Log CMV	< 1.70	Reference Range: < 1.70 Log IU
EBV EA IgG	12.1	Reference Range: < 9.0 U/mL
EBV Nuclear Antigen Antibody	191.0	Reference Range: < 18.0 U/mL
EBV VCA IgG	228.0	Reference Range: < 18.0 U/mL
EBV VCA IgM	< 10.0	Reference Range: < 36.0
EBV DNA PCR Qualitative	Detected	Reference Range: Not Detected
Epstein-Barr Virus DNA Quantitative PCR	495024	Reference Range: < 500 IU/mL
Log Epstein-Barr Virus DNA	5.69	< 2.70 Log IU

Additionally, an acute viral hepatitis panel was drawn, shown in Table 2.

**Table 2:** Acute Viral Hepatitis Panel, along with HIV Ab/Ag

Acute Viral Hepatitis Panel		
Hepatitis A	Result	Reference Range
Hepatitis A Antibody	Negative	Negative
Hepatitis B		
Hepatitis B Surface Ag	Negative	Negative
Hepatitis B Core IgM	Negative	Negative
Hepatitis C		
HCV Antibody	Negative	Negative
HIV Combo AB/AG	Nonreactive	Nonreactive

A panel that was drawn for aggressive B-cell lymphoma was positive for IGH/MYC gene rearrangement in 180/200 nuclei (90%) consistent with the t(8;14) (q24;q32) translocation: this finding is typically observed in NHL, especially Burkitt’s lymphoma. An evaluation for double-hit lymphoma was suggested and so, a second panel was drawn for a molecular cytogenetic diagnosis. This sample was negative for MYC gene rearrangement, a finding that essentially ruled out double-hit lymphoma. This was an important discovery as double-hit lymphoma is an aggressive subtype of B-cell NHL [9]; it gave us the ability to guide future prognosis and therapy.

On Day 7 of the patient’s latest admission, a lumbar puncture was determined to be negative; cerebrospinal fluid (CSF) cultures showed no organisms and no epithelial cells. 

Finally, on Day 17, a flow cytometry of paracentesis fluid was found to be positive for A lambda monotypic B-cell population (CD10+) with increased forward scatter, consistent with a large cell size overall. The immunophenotypic findings were consistent with peritoneal fluid involvement by the patients previously diagnosed PTLD. Therefore, the final diagnosis was positive for malignancy.

## Discussion

Patients with PTLD require careful evaluation and monitoring, as initial therapy requires immediate reduction in the patient’s immunosuppressive regimen to the lowest tolerated level. While this method increases the chance of transplant rejection, the patient and their provider should discuss risk versus benefit, of which factors include the patient’s general health, function of the transplanted organ, and type of PTLD being treated. The four types of PTLD are plasmocytic hyperplastic PTLD, polymorphic PTLD, monomorphic PTLD, and the classic Hodgkin-type lymphoma [10]. 

While there is no universally accepted prognostic scoring system for PTLD, a large cohort of 107 patients were analyzed by the Mayo Clinic and three criteria were identified: poor performance status, monomorphic pathology, and graft organ involvement [11]. Our patient’s final diagnosis was large B-cell non-Hodgkin’s lymphoma secondary to PTLD. She fell into the monomorphic subtype, however, she displayed no signs of kidney involvement (creatinine levels remained at baseline), and her overall performance function improved with reduction of immunosuppressive therapy. At the point of discharge, the patient's immunosuppressive regimen (tacrolimus and prednisone) was being held, and azathioprine had been stopped in order to continue use of allopurinol for management of hyperuricemia.

Traditional treatment of PTLD involves therapy with rituximab, a monoclonal antibody directed against CD20 [11]. The drug targets antigens expressed on the surface of mature and immature B lymphocytes. When monotherapy is not successful, oftentimes seen in patients categorized as “high risk group” (i.e. EBV-negative PTLD), follow-up anthracycline-based chemotherapy (R-CHOP: rituximab, cyclophosphamide, doxorubicin, vincristine, prednisolone) has proven to be successful in achieving long-term disease-free survival [12]. 

One week after discharge, the patient consented for treatment with R-CHOP, delivered once every 21 days. Prednisone was increased to 100mg for 5 days in order to initiate chemotherapy treatment. On the fifth day, Prednisone was lowered to its original dose of 10mg daily, and has since been maintained. Due to the patient's initiation of chemotherapy for treatment of PTLD, her pre-hospitalization dose of tacrolimus has been resumed for long-term renal graft protection.

## Conclusions

This case report highlights the presentation of PTLD, as well as the importance of its early recognition and treatment. PTLD is not an uncommon complication of solid organ transplantation, however symptomology and potential risk factors vary. We hope this case report will further help physicians recognize its presence and familiarize themselves with its prognostic factors and treatment options. 
